# Hydroxychloroquine in rheumatic autoimmune disorders and beyond

**DOI:** 10.15252/emmm.202012476

**Published:** 2020-07-26

**Authors:** Eliise Laura Nirk, Fulvio Reggiori, Mario Mauthe

**Affiliations:** ^1^ Department of Biomedical Sciences of Cells and Systems University of Groningen University Medical Center Groningen Groningen The Netherlands

**Keywords:** calcium, chloroquine, cytokines, lysosome, toll‐like receptors, Immunology, Chemical Biology

## Abstract

Initially used as antimalarial drugs, hydroxychloroquine (HCQ) and, to a lesser extent, chloroquine (CQ) are currently being used to treat several diseases. Due to its cost‐effectiveness, safety and efficacy, HCQ is especially used in rheumatic autoimmune disorders (RADs), such as systemic lupus erythematosus, primary Sjögren's syndrome and rheumatoid arthritis. Despite this widespread use in the clinic, HCQ molecular modes of action are still not completely understood. By influencing several cellular pathways through different mechanisms, CQ and HCQ inhibit multiple endolysosomal functions, including autophagy, as well as endosomal Toll‐like receptor activation and calcium signalling. These effects alter several aspects of the immune system with the synergistic consequence of reducing pro‐inflammatory cytokine production and release, one of the most marked symptoms of RADs. Here, we review the current knowledge on the molecular modes of action of these drugs and the circumstances under which they trigger side effects. This is of particular importance as the therapeutic use of HCQ is expanding beyond the treatment of malaria and RADs.

## Graphical abstract

Hydroxychloroquine has been heavily discussed in the context of COVID19, but this anti‐malarial drug is primarily used in rheumatic autoimmune disorders (RADs). This comprehensive review recapitulates our knowledge on the mode of action of this drug in RADs, and on its potential applications and side effects.

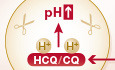

**Glossary**
Antigen‐presenting cells (APC): Cells that process proteins derived from pathogens or from dying/dead cells, into peptides that get presented on their surface, thereby activating T cells and initiating an immune response.
Autophagy: An intracellular process that delivers unwanted cytoplasmic material into lysosome for degradation.
B cells: A type of lymphocytes (white blood cells) that plays a crucial role in the adaptive immune response by producing antigen‐specific antibodies.
Calcium (Ca^2+^): Is the most abundant mineral in the human body and is vital for a multitude of cellular and physiological function. It is also an important second messenger in numerous signal transduction pathways.
Chloroquine (CQ)/hydroxychloroquine (HCQ): Originally developed to fight malaria, these drugs are used to treat rheumatic autoimmune diseases and are currently tested in clinical trials as therapies for other conditions.
Cytokines: Small secreted proteins that mediate communication and modulate interactions between cells, including immune cells.
Endosomes: Intracellular organelles that mainly function as a sorting and recycling hub for endocytosed and biosynthetic components, on their route to lysosomes.
Immune system: A network consisting of a variety of different cell types that defend the body against infections and other potentially harmful anomalies, and which, when misregulated, contributes or causes the development of an inflammatory disease.
Lysosome: Intracellular organelles containing a large battery of digestive enzymes that degrade extracellular and cytoplasmic material delivered to their interior by endocytosis and autophagy, respectively.
NADPH oxidase: A membrane‐bound multi‐subunit enzymatic complex at either the plasma or endosomal membrane, which participates in a variety of cellular functions, ranging from cellular signalling and gene expression to host defence mechanisms.
Primary Sjögren's syndrome: An autoimmune disease that belongs to the group of rheumatic autoimmune diseases, which affect saliva‐producing glands leading to symptoms such as dry mouth and dry eyes.
Retinopathy: Condition characterized by a damaged retina, which causes vision impairment, and is a documented adverse effect that can occur when taking HCQ and CQ.
Rheumatic autoimmune diseases: A group of conditions characterized by a dysregulated immune system, which primarily affect the muscles, joints, connective tissue and bones.
Systemic lupus erythematosus: An autoimmune disease that belongs to the group of rheumatic autoimmune diseases, which is the most common form of lupus and is associated with symptoms such as severe fatigue, joint pain and joint swelling.
T cells: A type of lymphocytes (white blood cells) that is a key component of the adaptive immune system and that orchestrates other cell types in response to antigens.
Toll‐like receptors (TLR): Transmembrane proteins that recognize specific molecules at either the plasma membrane or endosomes, and subsequently initiate signalling pathways that are crucial for the innate immune response.

## Introduction

Antimalarial drugs have a long history, starting around 400 years ago when quinine, a substance in the bark of the cinchona tree, was first used to fight *Plasmodium falciparum* infections (Woodward & Doering, 1945; Haładyj *et al*, 2018). CQ was the first potent and mass‐producible drug against malaria and was synthesized as an analogue of quinine (Shanks, 2016). Despite its remarkable antimalarial efficiency, CQ was deemed too toxic due to its side effects such as gastrointestinal and skin complications, retinopathy, cardiotoxicity or myopathy (Kalia & Dutz, 2007; Haładyj *et al*, 2018). The discovery of HCQ mitigated this issue, and HCQ is now regularly used in clinics under the brand name Plaquenil (Furst, 1996; Aviña‐Zubieta *et al*, 1998; Al‐Bari, 2014; Haładyj *et al*, 2018). Already during the Second World War, the positive effects of these two antimalarial drugs on RADs were observed. Soldiers taking CQ and HCQ as prophylaxis reported improvement of rashes and inflammatory arthritis. Today, CQ and particularly HCQ are commonly used to treat rheumatic and dermatological diseases, and are further being tested in clinical trials as potential drug candidates for COVID‐19, several types of cancer, diabetes type I and II, multiple sclerosis, recurrent miscarriages and myocardial infarction (Al‐Bari, 2014; clinicaltrials.gov).

RADs, such as systemic lupus erythematosus (SLE) (Ruiz‐Irastorza *et al*, 2010; Willis *et al*, 2012; Wu *et al*, 2017), rheumatoid arthritis (RA) (Khraishi & Singh, 1996) and primary Sjögren's syndrome (pSS) (Oxholm *et al*, 1998; Rihl *et al*, 2009; Kumar & Clark, 2012; Demarchi *et al*, 2017), are caused by a malfunctioning immune system that targets healthy tissues (Smith & Germolec, 1999) such as joints (Kumar & Clark, 2012). CQs and HCQs therapeutic role in RADs is linked to its anti‐inflammatory and immunomodulatory effects (Plantone & Koudriavtseva, 2018). These effects are achieved through the modulation of the autoimmune response by (i) impairing functions of the endolysosomal system through its lysosomotropic effects (Ziegler & Unanue, 1982; Kaufmann & Krise, 2007; Yoon *et al*, 2010), (ii) decreasing the levels of circulating pro‐inflammatory cytokines (Sperber *et al*, 1993; Van Den Borne *et al*, 1997), (iii) inhibiting T‐cell proliferation (Landewe *et al*, 1995; Costedoat‐Chalumeau *et al*, 2014), (iv) blocking Toll‐like receptors (TLRs) (Kyburz *et al*, 2006) and (v) autophagy inhibition (An *et al*, 2017c). However, numerous questions remain regarding both the mechanism of action of CQ and HCQ in RADs and the side effects caused by this compound.

In this review, we report on HCQ and CQ modes of action at the molecular and cellular levels in the context of RADs. Additionally, we discuss the relevance of these drugs in the treatment of cancer and infectious diseases. Finally, we summarize the side effects reported in patients taking HCQ for RADs and discuss how some of those can be explained by the current knowledge on CQ and HCQ.

## CQ and HCQ: modes of action

So far, CQ and HCQ have been reported to inhibit four sets of cellular functions: (i) endolysosomal activities, including autophagy; (ii) cytokine signalling, including endosomal Toll‐like receptor (TLRs); (iii) NADPH oxidase (NOX) signalling; and (iv) calcium (Ca^2+^) mobilization from the endoplasmic reticulum (ER). They might further modulate other cellular and organismal processes, e.g. Golgi trafficking (Mauthe *et al*, 2018), but the underlying mechanisms remain to be identified.

### Inhibition of lysosomal activity and autophagy

**Figure 1:**
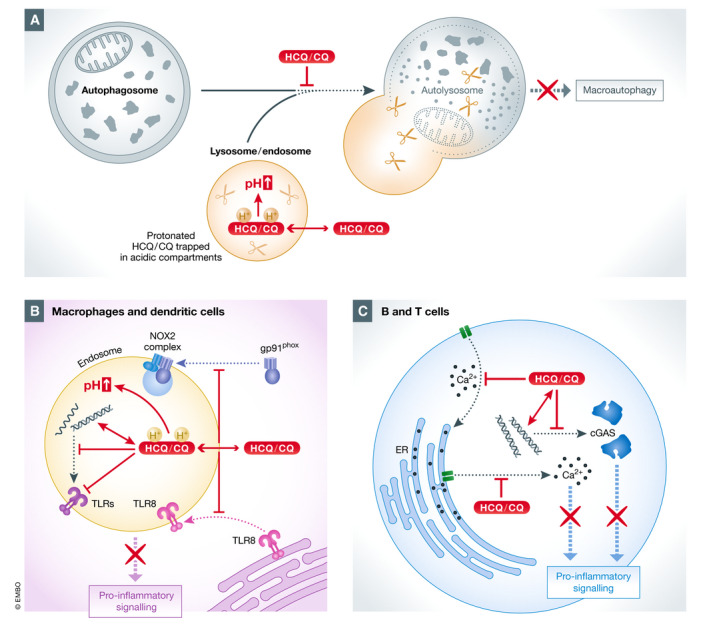
Molecular mechanisms of CQ and HCQ (A) CQ and HCQ are weak bases that accumulate inside acidic subcellular compartments, e.g. endosomes and lysosomes. They remain trapped in a protonated state, causing an increase of pH and thereby inhibiting the functions of these cellular compartments. Impairment of the autophagosome–lysosome fusion leads to autophagy inhibition. (B) CQ and HCQ alter endosomal TLR activation by increasing endosomal pH, by blocking the interaction between nucleic acids and endosomal TLRs (TRL3, TLR7 and TLR9) and by preventing translocation of TLR8 to endosomes. HCQ also blocks the correct assembly of the NOX2 complex by preventing the translocation of the NOX2 subunit gp91phox onto endosomes and consequently the formation of an active NOX2. (C) CQ and HCQ impair the release of Ca^2+^ from the ER, resulting in inhibition of Ca^2+^‐dependent signalling pathways. HCQ further inhibits the replenishing of intracellular Ca^2+^ stores from the extracellular space.

**Figure 2:**
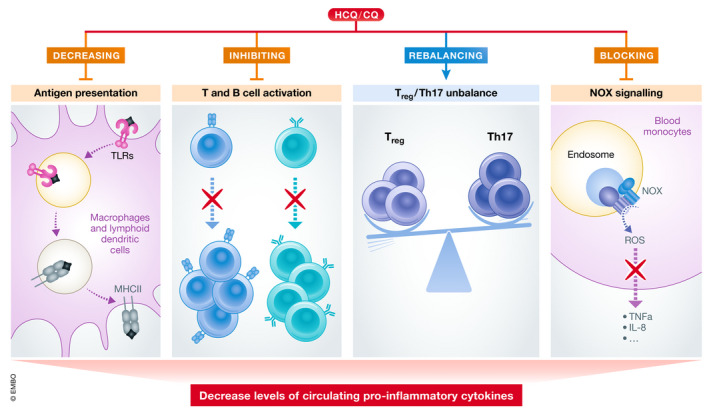
Effects of HCQ on the immune system At the cellular level, HCQ inhibits antigen presentation, B‐ and T‐cell activation and NOX signalling. In addition, it rebalances T_reg_/Th17 cell ratio. These multifaceted effects on different immune cells result in a decreased production and release of pro‐inflammatory cytokines.

CQ and HCQ are weak bases that easily cross cell membranes and accumulate in acidic subcellular compartments such as lysosomes and endosomes, where they remain trapped in a protonated state (Ohkuma & Poole, 1978). This leads to a pH increase in lysosomes from 4 to 6, causing inhibition of acidic proteases and other enzymes within the endolysosomal compartments (Fig 1A) (Ohkuma & Poole, 1978; Poole & Ohkuma, 1981; Ziegler & Unanue, 1982; Haładyj *et al*, 2018). As a result, antigen processing and subsequent presentation by MHC‐II complex on the cell surface of both macrophages and lymphoid dendritic cells are impaired (Guidos *et al*, 1984; Chesnut & Grey, 1985; Fox, 1993), dampening the adaptive immune response (Fig 2) (Fox, 1993). CQ and HCQ also increase pH levels within the Golgi stacks. This causes functional alterations of this organelle that possibly contribute to the cellular effects of these two drugs, e.g. by impairing transforming growth factor beta (TGF‐β) activity (Perkett *et al*, 2006; Rivinoja *et al*, 2009; Mauthe *et al*, 2018).

The ability to block lysosomal degradation also makes CQ and HCQ potent macroautophagy inhibitors (Fig 1A). Macroautophagy, hereafter called autophagy, is a conserved intracellular degradation pathway that is required to maintain cellular homeostasis by recycling damaged or unwanted cytoplasmic proteins, complexes and organelles (Eskelinen & Saftig, 2009). Autophagy plays a role in many physiological processes, and its misregulation is linked to pathologies such as cancer, neurodegeneration and inflammatory diseases (Mizushima *et al*, 2008; Levine *et al*, 2011; Dikic & Elazar, 2018; Levine & Kroemer, 2019). During autophagy, cytoplasmic cargoes are sequestered by double‐membrane vesicles called autophagosomes, which fuse with lysosomes to generate autolysosomes (Eskelinen & Saftig, 2009). Fusion with lysosomes and activity of the lysosomal enzymes are required to break down the autophagosomal cargoes and recycle the resulting metabolites. Impairment of both autophagosome–lysosome fusion and lysosomal degradative activity blocks autophagy (Klionsky *et al*, 2016). Although CQ and HCQ decrease the acidity of lysosomes (Seglen *et al*, 1979; Poole & Ohkuma, 1981; Mizushima *et al*, 2010), the primary inhibitory effect of these drugs on autophagy is blocking the fusion of autophagosomes and lysosomes, which is at least in part mediated by the dysregulation of the recruitment of specific SNARE proteins onto autophagosomes (Mauthe *et al*, 2018). This block results in an accumulation of autophagosomes in the cytoplasm (Mauthe *et al*, 2018), which can contribute to an enhanced autophagosome‐mediated signalling output (Martinez‐Lopez *et al*, 2013; Barrow‐McGee *et al*, 2016) and even compromise tumour cell viability (Button *et al*, 2017). Although HCQ and CQ have been extensively described as autophagy inhibitors, there is emerging evidence that these drugs induce a non‐canonical form of endocytosis (Florey *et al*, 2015; Jacquin *et al*, 2017).

### Inhibition of cytokine signalling

Activation of TLRs, especially in macrophages, monocytes and T helper cells, but also in neutrophils and endothelial cells, induces the production and secretion of pro‐inflammatory cytokines, a hallmark of RADs (Beutler & Cerami, 1989; Feldmann & Maini, 2001; Kim & Moudgil, 2017). Hence, inhibition of endosomal TLRs by HCQ or CQ is a powerful therapy approach for these diseases (Lafyatis *et al*, 2006). TLR9, activated by DNA in immune cells, can thus be inhibited by HCQ and CQ (Yi *et al*, 1998; Ahmad‐Nejad *et al*, 2002). TLR7, activated by guanosine analogues, can also be inhibited by CQ, but to a lesser extent than TLR9 (Lee *et al*, 2003), indicating different inhibitory mechanisms. TLR3 is mainly activated by poly(I‐C), but also by debris originating from necrotic synovial fluid cells in RA patients, and both modes of activation are hampered by HCQ and CQ (Brentano *et al*, 2005; Jolly *et al*, 2014; Imaizumi *et al*, 2017). In general, inhibition of TLR3, TLR7 and TLR9 by HCQ and CQ has been attributed to their ability to impair endosomal acidification (Macfarlane & Manzel, 1998; Lafyatis *et al*, 2006; Schrezenmeier & Dörner, 2020), as activation of endosomal TLRs and subsequent downstream signalling only takes place within acidified compartments (Fig 1B) (Blasius & Beutler, 2010).

Beside endosomal acidification, Kuznik and colleagues discovered a second mechanism by which CQ impairs TLR signalling. They showed that CQ could inhibit endosomal TLR signalling after stimulation with nucleic acids at concentration too low to influence the endosomal pH. Under those conditions, CQ blocks endosomal TLR activation by directly interacting with TLR ligands, such as nucleic acids, which changes the nucleic acid secondary structure and prevents their binding to endosomal TLRs (Macfarlane & Manzel, 1998; Kužnik *et al*, 2011). This notion is further supported by the observation that HCQ specifically blocks activation of dendritic cells and macrophages by DNA but not by LPS, although LPS also stimulates these cells via a signalling cascade emanating from endosomes (Häcker *et al*, 1998).

A third mechanism that interferes with inflammatory cytokine production is the ability to disrupt GMP‐AMP synthase (cGAS) signalling (An *et al*, 2015, 2018). cGAS is a crucial component of the cGAS–stimulator of interferon gamma (IFN) genes (STING) signalling cascade that is required for the IFN type I response in immune cells (Sun *et al*, 2013), making it an important player in activation of pro‐inflammatory response in autoimmune diseases (Gao *et al*, 2015; Kato *et al*, 2018). cGAS is also upregulated in a portion of SLE patients (An *et al*, 2017a,b), and interestingly, HCQ and CQ can inhibit cGAS binding to its ligands, e.g. DNA, *in vitro* and in a T‐cell line (An *et al*, 2015). Importantly, inhibition of cGAS activation results in reduced IFNβ expression (An *et al*, 2015) (Fig 1C).

### Inhibition of NADPH oxidase

NOX is a protein complex involved in numerous pro‐inflammatory signalling cascades, such as tumour necrosis factor alpha (TNFα)‐ and interleukin (IL)‐1β‐induced cascades. Activation of endosomal NOX, which leads to the generation of reactive oxygen species (ROS), requires the endocytic internalization and delivery to endosomes of cell surface ligand–receptor complexes (Müller‐Calleja *et al*, 2017). HCQ blocks the NOX‐mediated signalling cascades triggered by TNFα and IL‐1β in monocytes by blocking translocation of gp91phox, the catalytic subunit of NOX, from the cytosol onto endosomal membranes without changing the endosomal pH (Müller‐Calleja *et al*, 2017). This inhibition prevents the correct assembly and activation of NOX, hindering the downstream cellular events and the production of the pro‐inflammatory cytokines TNFα and IL‐8. HCQ also prevents the redistribution of TLR8 from the ER to endosomes, which is necessary to mediate the inflammatory response (Müller‐Calleja *et al*, 2017) (Fig 1B).

### Inhibition of Ca^2+^ signalling

Ca^2+^ mobilization from both the ER and extracellular space into the cytoplasm and subsequent Ca^2+^‐dependent signalling is an important mechanism to activate cells of the immune system, such as T and B cells (Feske, 2007). High cytoplasmic levels of Ca^2+^ act as a second messenger for the activation of signalling pathways and transcription factors that regulate the expression and secretion of cytokines and other immune regulatory factors (Izquierdo *et al*, 2014). Ca^2+^ release from the ER can be impaired by HCQ (Goldman *et al*, 2000; Xu *et al*, 2015; Wu *et al*, 2017), leading to the inhibition of intracellular signals. In particular, T‐cell and B‐cell receptor‐mediated intracellular Ca^2+^ mobilization from both intracellular stores and the extracellular milieu is inhibited by HCQ in a dose‐dependent manner (Goldman *et al*, 2000). This impairment of Ca^2+^ mobilization is at least partially caused by the reduction of the Ca^2+^ stored intracellularly and the inability to replenish these intracellular stores with extracellular Ca^2+^ (Goldman *et al*, 2000). This further enhances its negative impact on the Ca^2+^‐dependent signalling pathways (Fig 1C) (Feske, 2007). The precise mechanism of HCQ‐induced reduction of internal Ca^2+^ mobilization remains unknown. However, it has been shown that HCQ does not reduce the availability of inositol 1,4,5‐trisphosphate, but rather the binding to its intracellular receptors that promotes Ca^2+^ release (Misra *et al*, 1997).

## The impact of CQ and HCQ on the immune system in autoimmunity

Autoimmunity is characterized by an overreaction of the immune system (Smith & Germolec, 1999), which is linked to both innate and adaptive immunity (Mescher, 2016). The innate immune system is responsible for the initial recognition of pathogens, which is mostly carried out by antigen‐presenting cells (APCs), e.g. dendritic cells, and eventually triggers the activation of the adaptive immune system (Mescher, 2016). In particular, when APCs get directly activated through exposure to pathogen‐associated molecular patterns, they initiate both cell‐ and antibody‐mediated immune responses, which are mediated by the T and B cells, respectively (Christmas, 2010). The cell‐mediated response is executed by T cells that get activated by APCs through antigen presentation at their surface via MHC molecules. In contrast, B cells are activated through T helper (Th) cells and cytokines that are secreted by APCs (Mescher, 2016). Activated B cells produce and secrete additional pro‐inflammatory cytokines and antibodies to further stimulate the immune reaction (Mescher, 2016).

HCQ and CQ negatively regulate many aspects of these innate and adaptive immune responses by reducing inflammation, and ultimately the severity of autoimmune diseases (Fig 2).

### Inhibition of pro‐inflammatory cytokine secretion

Through the inhibition of endosomal TLR signalling, HCQ and CQ treatment decreases the levels of pro‐inflammatory cytokines produced by peripheral mononuclear cells in the blood, including IFNγ (Van Den Borne *et al*, 1997), TNFα (Picot *et al*, 1991; Van Den Borne *et al*, 1997; Jang *et al*, 2006), IL‐1 (Picot *et al*, 1991; Sperber *et al*, 1993; Jang *et al*, 2006), IL‐6 (Sperber *et al*, 1993; Van Den Borne *et al*, 1997; Jang *et al*, 2006) and IL‐2 (Landewe *et al*, 1995). The reduction of TLR signalling‐mediated activation of immune cells by both drugs consequently decreases the aberrant immune response and diminishes inflammation symptoms observed in rheumatic patients (da Silva *et al*, 2013). In addition to directly inhibiting endosomal TLR signalling, CQ and HCQ can interfere with the intracellular signals that lead to both the release of phorbol ester‐induced arachidonic acid and the block of pro‐inflammatory cytokines secretion (e.g. TNFα and IL‐1) in mouse macrophages (Bondeson & Sundler, 1998). In particular, activation of phospholipase A2 by phorbol esters, but not by Ca^2+^, is inhibited by HCQ and CQ, which blocks the synthesis of arachidonic acid. Furthermore, these compounds negatively impact the generation of zymosan‐induced formation of inositol phosphates, a product of phospholipase C activity (Matsuzawa & Hostetler, 1980), suggesting that they have an inhibitory effect on this enzyme as well (Bondeson & Sundler, 1998). HCQ also inhibits Ca^2+^‐activated K^+^ channels in macrophages, and consequently K^+^ efflux, which could result in impaired inflammasome activation and pro‐inflammatory cytokine release (Eugenia Schroeder *et al*, 2017).

High levels of pro‐inflammatory cytokines are a central characteristic of the RA pathogenesis (McInnes & Schett, 2007; Blasius & Beutler, 2010; Pollard *et al*, 2013; Schinnerling *et al*, 2017; Muskardin & Niewold, 2018). In particular, stimulatory cytokines (i.e. IL‐1, IL‐6, IL‐12, IL‐15, IL‐17, IL‐23 and type I and II IFN for T cells, and B‐cell activating factor (BAFF) for B cells) activate T and B cells, which in turn produce pro‐inflammatory cytokines and autoantibodies, respectively. Pro‐inflammatory cytokines contribute to RA pathogenesis by promoting autoimmunity, maintaining chronic inflammatory synovitis and stimulating the destruction of joint tissues. They also play a role in the maturation and activation of osteoclasts, the cells responsible for breaking down bone tissue (McInnes & Schett, 2007).

Excessive production of BAFF, a cytokine essential for B‐cell physiology, alters the immune tolerance by contributing to the maturation and survival of self‐reactive B cells, the major source for autoantibodies contributing to joint inflammation (Mahdy *et al*, 2014). Reduction of the high BAFF levels in the serum from RA patients by HCQ (Mahdy *et al*, 2014) improves symptoms of RADs, both in animal models and in clinical trials (Sun *et al*, 2008).

Cytokines like BAFF, TNFα, IFNα and IFNγ are also major contributors to SLE severity, by promoting B‐cell survival and autoantibody production, and contributing to organ inflammation (Rönnblom & Elkon, 2010). Thus, the modulation of their levels represents a potential therapeutic avenue (Rönnblom & Elkon, 2010). This is supported by a cohort study showing that treatment of SLE patients with HCQ results in a decrease of type I IFN levels and concomitant reduction of disease severity (Willis *et al*, 2012). HCQ can also directly affect the production of autoantibodies by B cells through TLR9 inhibition. Particularly, HCQ interferes with the differentiation of memory B cells into antibody‐producing plasmablasts, a subset of B cells, by inhibiting TLR9 activation (Torigoe *et al*, 2018).

Although the pathogenesis of pSS is not fully understood yet, activation of exocrine gland epithelium cells is thought to lead to the release of pro‐inflammatory cytokines such as IFNα and IFNβ (both type I IFN), IL‐7 and BAFF, and chemokines (Retamozo *et al*, 2018). These factors stimulate further activation of APCs, but also of T and B cells, which promotes inflammation and autoimmunity (Retamozo *et al*, 2018). Only a few studies investigated HCQ administration in pSS patients. Nonetheless, pSS patients treated with HCQ have a significant lower BAFF levels in the serum, and an improvement in saliva production (Mumcu *et al*, 2013), indicating that this drug might be a promising therapy for pSS as well.

### Inhibition of B‐ and T‐cell activation through Ca^2+^ signalling

Through T‐cell receptors (TCRs) on their surface, T cells recognize antigens that are presented by APCs and get activated (Goldman *et al*, 2000). This results in both their proliferation and the release of various cytokines, including IL‐6 and TNFα (Sperber *et al*, 1993). One important step in the signalling cascade downstream of TCRs is the increase of intracellular Ca^2+^ levels, which is released from internal Ca^2+^ storages such as the ER. As previously mentioned, HCQ can impair the release of Ca^2+^ from the ER, which consequently inhibits T‐cell activation (Goldman *et al*, 2000; Xu *et al*, 2015; Schmidt *et al*, 2017). HCQ also negatively influences the expression and activity of CD154 on T cells, which is needed for B‐cell activation (Wu *et al*, 2017; Dewitte *et al*, 2020). CD154 expression is controlled by the nuclear factor of activated T cells (NFAT), a transcription factor that relies on Ca^2+^ release from the ER (Wu *et al*, 2017). By impairing this event, HCQ inhibits NFAT nuclear translocation, resulting in decreased gene expression of CD154 (Wu *et al*, 2017). Altogether, these studies show that blocking Ca^2+^ release from the ER by HCQ leads to a multilevel inhibition of T‐ and B‐cell activation, thereby hindering the immune response (Fig 2).

### Modulation of Th17 and T_reg_ populations

Alterations in autophagic activity play an important role in the pathophysiology of T‐ and B‐cell‐mediated autoimmunity (Weindel *et al*, 2015; van Loosdregt *et al*, 2016; Alessandri *et al*, 2017; Mocholi *et al*, 2018; Zhang *et al*, 2019). In this context, autophagy is required to maintain cellular homeostasis in T cells (An *et al*, 2017c) and autophagy deficiency impairs MHC class II presentation and contributes to the generation of autoreactive T cells by thymic epithelial cells (Levine *et al*, 2011). Moreover, plasma cells require autophagy to sustain immunoglobulin production and B‐cell development (Wu & Adamopoulos, 2017). An imbalance within the T‐cell populations, more specifically an increase in the number of Th17 cells and a decrease in that of T_reg_ cells, has been linked to pathogenesis of autoimmune diseases (Yang *et al*, 2011a; Jadidi‐Niaragh & Mirshafiey, 2012; Álvarez‐Rodríguez *et al*, 2019), including SLE (An *et al*, 2017c; Álvarez‐Rodríguez *et al*, 2019). This imbalance leads to an increased secretion of pro‐inflammatory cytokines such as IL‐17 and IL‐6, and a reduction of the levels of circulating factors like TGF‐β, which suppresses inflammation and autoimmunity (An *et al*, 2017c; Geng *et al*, 2020). This latter effect can be dampened with HCQ and CQ, as those drugs rebalance the Th17/T_reg_ ratio (An *et al*, 2017c; Yang *et al*, 2018; Álvarez‐Rodríguez *et al*, 2019; Park *et al*, 2019; Geng *et al*, 2020). Mechanistically, this could be caused by an alteration of autophagy, as an induction of this process is observed in SLE patients (An *et al*, 2017c). Thus, An and colleagues thought to suppress hyperactivated autophagy by administrating HCQ to lupus MLR/pr mice, an animal model for SLE. In addition to lowering autophagic activity in this model, HCQ rebalanced Th17 and T_reg_ cell numbers, which led to a decrease in pro‐inflammatory cytokine levels (Fig 2) and a concomitant augmentation of anti‐inflammatory cytokines, resulting in the suppression of the autoimmune response (An *et al*, 2017c). Moreover, CQ positively regulates T_reg_ differentiation by stimulating transcriptional activity of Nurr1 and FOXP3, while simultaneously suppressing Th17 differentiation and gene expression (Álvarez‐Rodríguez *et al*, 2019; Park *et al*, 2019). More evidence that Th17 cells play a central role in RA and SLE pathogenesis comes from the detection of IL‐6, IL‐17 and IL‐22 in synovial fluids from patients suffering from those diseases (Lubberts *et al*, 2005; da Silva *et al*, 2013). High levels of these cytokines correlate with synovial inflammation, T‐cell activation and the osteoclast activity upregulation causing bone erosion (da Silva *et al*, 2013). Administration of HCQ reduces Th17 cell activation and consequently production of IL‐6, IL‐17 and IL‐22 (da Silva *et al*, 2013; Yang *et al*, 2018).

### Impact of NOX inhibition on the immune system

NOX inhibition by HCQ impairs the production of pro‐inflammatory cytokines and the correct distribution of TLR8, thereby dampening the immune response (Müller‐Calleja *et al*, 2017). This inhibition also positively affects nitric oxide (NO) bioavailability (Gómez‐Guzmán *et al*, 2014). NO is involved in a multitude of physiologic functions, including the regulation of blood vessel tone and vasodilation, and is rapidly inactivated by ROS (Nagy *et al*, 2010). In SLE patients, NO bioavailability is severely lowered by high ROS levels, particularly O^2−^, resulting in endothelial dysfunction (Griendling & Alexander, 1997; Landmesser & Harrison, 2001; Gómez‐Guzmán *et al*, 2014). By blocking NOX, the major producer of O^2−^ in the vascular wall, HCQ treatment reduces ROS levels and helps to prevent endothelial dysfunction in a mouse model for SLE (Gómez‐Guzmán *et al*, 2014). In agreement with this concept, NOX inhibition by HCQ reduces thrombus formation, which is a well‐known clinical manifestation in SLE, in a venous thrombus mouse model (Müller‐Calleja *et al*, 2017; Miranda *et al*, 2019) (Fig 2).

Thus, at the cellular level, HCQ and CQ inhibit antigen presentation, NOX signalling, B‐ and T‐cell activation, and rebalance T_reg_/Th17 cell ratio. These multifaceted effects on different immune cells synergistically result in a decreased production and release of pro‐inflammatory cytokines, a common hallmark of RADs (Fig 2).

## Clinical impact of HCQ on RADs

HCQ is administered orally in tablet form as hydroxychloroquine sulphate (Pastick *et al*, 2020). It is absorbed in the gastrointestinal tract (Mclachlan *et al*, 1994) before being widely distributed throughout the body to muscles, liver, spleen, lungs, kidneys, pituitary and adrenal glands, and tissues that contain melanin (Haładyj *et al*, 2018). Daily dosage of HCQ ranges from 200 to 600 mg for RADs, from 200 to 400 mg for dermatological disorders (Ben‐Zvi *et al*, 2012), from 200 to 1,200 mg in cancers (Chude & Amaravadi, 2017) and from 200 to 800 mg for various infectious diseases. Its half‐life in the body ranges between 40 and 50 days (Mclachlan *et al*, 1994), and 30–40% of HCQ is protein‐bound (Furst, 1996), resulting in 60–70% unbound, pharmacologically active drug (Rang *et al*, 2016). The majority of HCQ is excreted through the kidneys, while the rest is metabolized by the liver or excreted through faeces (Furst *et al*, 1999; Haładyj *et al*, 2018). Contraindications for taking HCQ are a history of retinopathy or visual field changes, hypersensitivity to 4‐aminoquinoline compounds and long‐term therapies in children (https://www.fda.gov/). HCQ is, however, considered safe during pregnancy (Kaplan *et al*, 2016; Haładyj *et al*, 2018).

**Figure 3:**
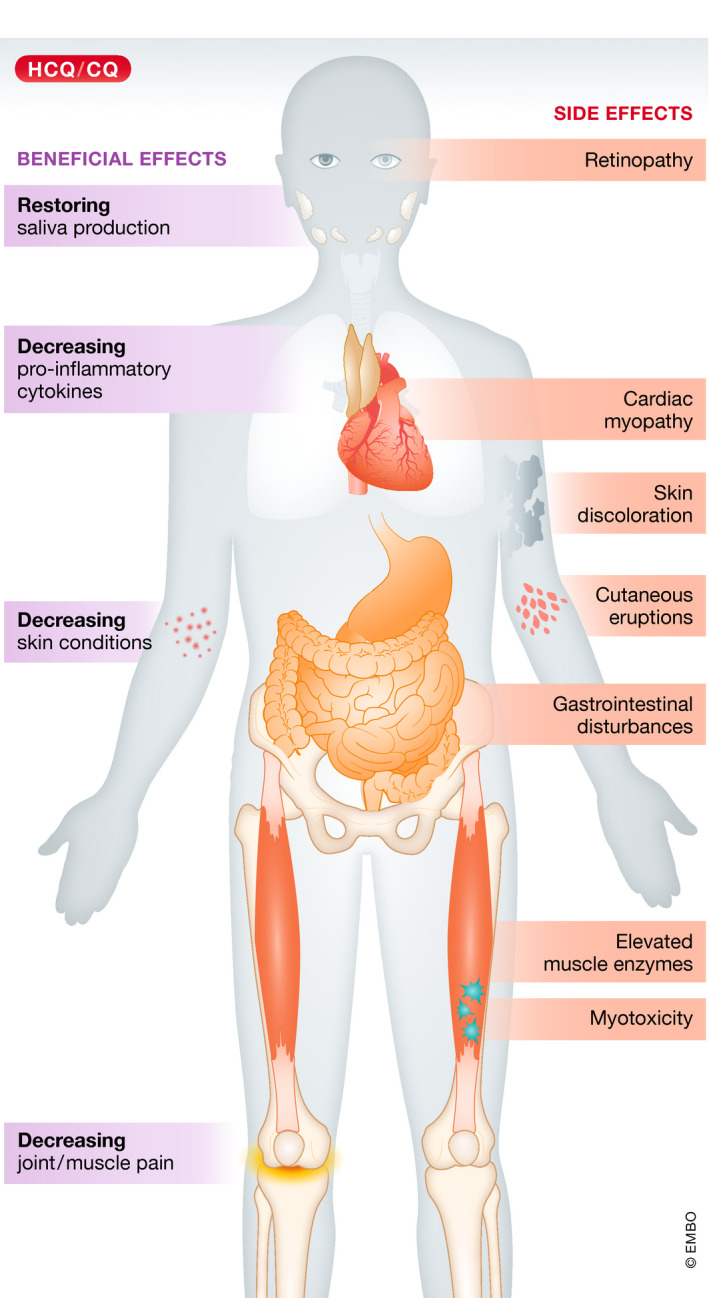
Beneficial and side effects caused by HCQ in RAD patients In RADs, HCQ treatment predominantly alleviates the symptoms (purple boxes) by inhibiting the production and release of pro‐inflammatory cytokines. As a consequence, HCQ diminishes skin conditions. There are also indications that HCQ both decreases cartilage degradation and consequently reduces joint and muscle pain, and helps to restore saliva production. Usage of HCQ can cause side effects (orange boxes); the most common are gastrointestinal disturbances, skin discoloration, cutaneous eruptions and elevated muscle enzymes, whereas retinopathy, cardiac myopathy and myotoxicity are rare, but severe.

HCQ ameliorates classical RAD symptoms, such as skin problems and joint pain, predominantly by decreasing the inflammation reaction in patients (Fig 3). In SLE, HCQ is given to patients as either a single or a combinatorial therapy together with steroids and immunosuppressive drugs, to improve patients’ life expectancy by reducing lupus flares and accrual of organ damage (Ponticelli & Moroni, 2017). Case studies have revealed that HCQ treatment reduces SLE symptoms and improves long‐term survival of patients, while individuals not treated with HCQ have an increased risk of severe SLE exacerbations (James *et al*, 2007; Ruiz‐Irastorza *et al*, 2010; Willis *et al*, 2012).

Similarly, HCQ treatment produces significant clinical improvement and functional capacity in RA patients (Smolen *et al*, 2014; Haładyj *et al*, 2018). In RA, prevention of cartilage degradation, which causes joint destruction, is an important aspect of the therapeutic approach (Kumar & Clark, 2012). Cartilage degradation is mostly caused by pro‐inflammatory cytokines, such as IL‐1, IL‐17 and TNFα, and their production can be repressed by HCQ treatment (Picot *et al*, 1991; Sperber *et al*, 1993; Van Den Borne *et al*, 1997; Jang *et al*, 2006; McInnes & Schett, 2007; da Silva *et al*, 2013). *In vitro* experiments have also established that CQ inhibits proteoglycan turnover (Fulkerson *et al*, 1979; Ackerman *et al*, 1981; Schug & Kalbhen, 1995; Rainsford *et al*, 2015), and early autoradiographic studies following tritium‐labelled HCQ have revealed that this drug accumulates in the cartilage of mice (Cecchi & Porzio, 1964). These findings and its water‐soluble properties led to the proposition that HCQ accumulates in the cartilage by binding acidic proteoglycans and protecting them from degradation by proteolytic enzymes (Rainsford *et al*, 2015). Although an early study pointed out that CQ and HCQ can indeed inhibit cartilage breakdown, slowing down the disease progression and preventing further joint damage in RA patients (Julkunen *et al*, 1976), more recent investigations could not confirm a positive effects on joint damage (Sanders, 2000; Smolen *et al*, 2014; Haładyj *et al*, 2018).

The therapeutic benefits of HCQ administration on pSS classical symptoms, e.g. sicca symptoms, remain controversial; some studies documented beneficial effects (Tishler *et al*, 1999; Rihl *et al*, 2009; Yavuz *et al*, 2011; Mumcu *et al*, 2013), while others reported none (Gottenberg *et al*, 2014; Yoon *et al*, 2016; Wang *et al*, 2017). HCQ treatment, however, ameliorates extraglandular symptoms (Fox *et al*, 1996; Demarchi *et al*, 2017), and according to the Sjögren's Syndrome Foundation's clinical practice guidelines (https://www.sjogrens.org/), disease‐modifying anti‐rheumatic drugs are recommended to treat musculoskeletal pain, with HCQ being the therapeutic approach of choice (Carsons *et al*, 2015). HCQ also reduces immunological alterations of pSS, such as decreased levels of immunoglobulins, erythrocyte sedimentation rate, serology and IL‐6 production (Tishler *et al*, 1999; Yavuz *et al*, 2011; Mumcu *et al*, 2013). Furthermore, in a retrospective analysis, HCQ administration to pSS patients significantly improved saliva production (Rihl *et al*, 2009). This improvement was more pronounced in patients who were positive for autoantibodies against anti‐α‐fodrin, an intracellular filamentous cytoskeleton protein. While the cause for this difference remains unknown, a possible explanation is that HCQ could improve saliva production by decreasing elevated levels of cholinesterase, an enzyme that counteracts saliva production (Dawson *et al*, 2005).

## HCQ and CQ in non‐rheumatologic diseases

### Anti‐viral effects

The anti‐viral function of HCQ and CQ has mainly been linked to their ability to increase the pH of the endosomal system and the trans‐Golgi network (TGN) (Savarino *et al*, 2003). Thus, these drugs are able to inhibit cell entry of numerous viruses, as a low endosomal pH is required for the fusion of endocytosed virions with the limiting membrane of endosomes. In this context, CQ and HCQ decrease replication of viruses such as dengue virus (DENV2), chikungunya virus, hepatitis A and C virus, influenza A virus, Zika virus, severe acute respiratory syndrome coronavirus (SARS‐CoV) and Borna disease virus in cellular models (Bishop, 1998; Gonzalez‐Dunia *et al*, 1998; Keyaerts *et al*, 2004; Vincent *et al*, 2005; Blanchard *et al*, 2006; De Clercq, 2006; Eng *et al*, 2006; Di Trani *et al*, 2007; Sourisseau *et al*, 2007; Khan *et al*, 2010; Ashfaq *et al*, 2011; Boonyasuppayakorn *et al*, 2014; Farias *et al*, 2015; Delvecchio *et al*, 2016; Shiryaev *et al*, 2017). For some viral structural proteins, a maturation step involving post‐translational modification and/or processing in the TGN is crucial for their function and ultimately for the assembly of infectious viral particles, e.g. glycosylation of HIV gp120 (Tsai *et al*, 1990; Savarino *et al*, 2004) or cleavage of the DENV2 prM protein (Randolph *et al*, 1990). Glycosylation in the TGN is also required for the correct assembly of ACE2, the entry receptor for SARS‐CoV (Vincent *et al*, 2005). Thus, HCQ and CQ contribute to inhibit viral infections by neutralizing the pH of intracellular organelles, interfering with important processes required for viral life cycle.

Although HCQ and CQ have shown beneficial therapeutic effects in animal models for DENV2, hepatitis C virus, avian influenza A virus, Zika virus and SARS‐CoV infections, clinical trials have so far failed to conclusively prove their anti‐viral potential in humans (Rodrigo *et al*, 2020; Fragkou *et al*, 2020; McKee *et al*, 2020). This might be due to the fact that drug concentrations required to de‐acidify intracellular compartments cannot easily be reached in humans (Al‐Bari, 2017). Therefore, neither HCQ nor CQ is currently recommended as anti‐viral drugs (Rodrigo *et al*, 2020). During the SARS‐CoV‐2 pandemic in 2020, the need to find an effective medication has brought major attention to HCQ and CQ due to their ability to both inhibit viral infections and dampen the massive cytokine response that is observed in SARS‐CoV‐2‐infected patients (Badgujar *et al*, 2020; Ibáñez *et al*, 2020; Moore & June, 2020). The effectiveness of HCQ and CQ against SARS‐CoV‐2, however, has so far not been proven in humans, and the results at the time that this review was completed were still controversial (Boulware *et al*, 2020; Fragkou *et al*, 2020).

### Anti‐cancer therapy

CQ and HCQ are being increasingly used in clinical trials to treat cancer (https://clinicaltrials.gov/). Because high doses are required to achieve anti‐tumoural effects in monotherapies, they are often used in combination with radiotherapy and/or other chemotherapeutical drugs (Plantone & Koudriavtseva, 2018). We briefly discuss here possible mechanisms of action for HCQ and CQ in cancer. For a more detailed discussion on this topic, more specific reviews are available (Manic *et al*, 2014; Pascolo, 2016; Levy *et al*, 2017; Shi *et al*, 2017; Verbaanderd *et al*, 2017).

Elevated autophagic activity is crucial for tumour cell survival and growth as it supplies the high demand of nutrients within a developed tumour (Amaravadi *et al*, 2016). This is especially relevant for autophagy‐dependent cancers that rely on this pathway when faced with metabolic stress. Consequently, HCQ or CQ treatment has been successful in regressing the growth of some of those cancers in preclinical studies (e.g. with RAS pathway mutations (Guo *et al*, 2011; Lock *et al*, 2011), such as specific pancreatic cancers (Mancias & Kimmelman, 2011; Yang *et al*, 2011b; Sousa *et al*, 2016), or BRAF‐driven tumours (Levy *et al*, 2014; Strohecker *et al*, 2013; Xie *et al*, 2015). The effectiveness of HCQ and CQ in cancer therapy is, however, controversial. In animal models, HCQ dosages are often 50 mg/kg/day or higher, which is too high to be administered in humans (Pascolo, 2016), and with lower dosages, autophagy is not sufficiently inhibited to achieve tumour regression (Pascolo, 2016). Moreover, some cancer cells (e.g. derived from breast tumours or melanomas or KRAS‐driven cancer cell lines) have shown CQ‐mediated cell growth inhibition that was independent of autophagy (Maycotte *et al*, 2012; Maes *et al*, 2014; Eng *et al*, 2016).

Various cancer cells express high levels of TLR9, e.g. breast and prostate cancer cells (Merrell *et al*, 2006; Verbaanderd *et al*, 2017), which is linked to cancer invasiveness *in vitro* and associated with poor prognosis (Väisänen *et al*, 2013; Verbaanderd *et al*, 2017). TLR9‐mediated NF‐κB signalling is required for cancer cell migration and proliferation in gastric cancer cell models, which is inhibited by CQ (Zhang *et al*, 2015). The exact molecular mechanism of TLR9 signalling inhibition in cancer cells remains unknown.

Another mechanism by which HCQ affects cancer growth is by modulating the immune system. Tumour‐associated macrophages (TAMs), which are phenotypically described as M2 macrophages, play a role in promoting tumour growth and immune escape, angiogenesis and metastasis (Mantovani *et al*, 2017; Li *et al*, 2018). In contrast, tumour killing macrophages (M1 macrophages) have an opposite effect and are activated by cytokines such as IFNγ, which are released from T cells (De Palma & Lewis, 2013; Ostuni *et al*, 2015). Interestingly, in a melanoma‐bearing mouse model, intraperitoneal injection of 75 mg/kg CQ effectively inhibited melanoma growth in a T‐cell‐dependent manner, and prolonged animal survival (Chen *et al*, 2018). Mechanistically, CQ can switch TAMs into M1 macrophages by raising lysosomal pH, and thereby mobilizing lysosomal Ca^2+^ through upregulation of the lysosomal Ca^2+^ channel MUCOLIPIN1. The release of lysosomal Ca^2+^ then activates the p38 and NF‐κB pathways, but also the transcription factor EB, resulting in an enhanced anti‐tumour T‐cell response (Chen *et al*, 2018). By stimulating the T‐cell‐mediated immune response and simultaneously decreasing immune inhibitory cells, including TAMs and T_regs_, and cytokines such as TGF‐β and IL‐10, CQ treatment reduced breast cancer growth and prolonged mice survival in a breast xenograft model (Zhang *et al*, 2017). Another important aspect of anti‐cancer immunity is the activation of immune cells by sensing danger signals (e.g. HMGB1). Danger signals are subsequently recognized by receptors, such as TLR4 on dendritic cells (Apetoh *et al*, 2007). One function of TLR4 is to preserve engulfed tumour antigens from enhanced degradation, and thereby favour antigen presentation. The loss of antigen presentation capacity in TLR4‐deficient dendritic cells can be restored by CQ, possibly by raising lysosomal pH, which contributed to tumour size reduction in a *tlr4* ^−/−^ thymoma mouse model (Apetoh *et al*, 2007). Along these lines, CQ reduced breast cancer growth in mice after irradiation by enhancing apoptotic and immunogenic tumour cell death (Ratikan *et al*, 2013). The enhanced immune response was attributed to a decreased degradation of tumour antigens in dendritic cells, resulting in an increased antigen presentation (Ratikan *et al*, 2013).

HCQ and CQ can also inhibit CXCL12/CXCR4 signalling, which is involved in chemotaxis and adhesion of tumour cells and of growth factors secretion that are key for cancer progression (Sun *et al*, 2010; Kim *et al*, 2012; Verbaanderd *et al*, 2017). Moreover, HCQ and CQ interfere with the activation of growth‐promoting pathways in cancer stem cells, thereby suppressing the regrowth of tumours (Li *et al*, 2008; Balic *et al*, 2014; Choi *et al*, 2014).

Multiple reports further describe the mechanisms by which CQ triggers cell death in tumour cells. CQ induces apoptosis of cancer cells by either stimulating the mitochondrial apoptotic pathway (Du Jiang *et al*, 2010) or activating the p53‐dependent transcription of pro‐apoptotic genes (Zhou *et al*, 2002; Loehberg *et al*, 2007, 2012; Maclean *et al*, 2008; Kim *et al*, 2010; Bieging *et al*, 2014). Additionally, several studies have suggested that CQ intercalates into DNA and disturbs chromatin topology (O'Brien *et al*, 1966; Sternglanz *et al*, 1969; Field *et al*, 1978; Yin *et al*, 2003), which could lead to an impairment in DNA repair mechanisms, and in turn cause DNA damage and enhance cell death (Michael & Williams, 1974; Liang *et al*, 2016; Weyerhäuser *et al*, 2018).

Besides directly targeting tumour cells, CQ also affects tumour angiogenesis by altering endothelial cell functionality. CQ administration leads to NOTCH1 accumulation in endothelial cell endosomes, stimulating the downstream signalling that leads to tumour vessel normalization, and resulting in reduced tumour invasion and metastasis (Maes *et al*, 2014). Therefore, CQ also improves the delivery and efficacy of other chemotherapeutics (Maes *et al*, 2014).

HCQ and CQ thus show potential in inhibiting tumour growth and modulating tumour immune response through various mechanisms. It is, however, important to reiterate that the doses used to achieve relevant effects in cancer therapies are often substantially higher than the doses used to treat RADs. Moreover, when treating cancer or viral infections, one has to keep in mind that HCQ and CQ also have immune suppressive functions that could negatively influence its beneficial effect for the patients.

## Side effects of HCQ in RADs

Side effects of HCQ treatment are rare, but nonetheless exist, and can be very serious, especially during prolonged administration (Haładyj *et al*, 2018). In Table EV1, we provide a comprehensive overview of the known side effects caused by HCQ in RADs and their prevalence. Overall, the most common side effects in RAD patients taking HCQ or CQ are gastrointestinal disturbances, skin discolorations, cutaneous eruptions and elevated muscle enzymes. Although rare, retinopathy, neuromuscular and cardiac toxicities (Fig 3) are the most serious and life‐threatening side effects potentially triggered by HCQ (Plantone & Koudriavtseva, 2018).

### Retinopathy

Prolonged administration of HCQ or CQ can cause retinopathy and loss of retinal function that, when ignored, can result in permanent vision loss (Jorge *et al*, 2018). The primary site of toxicity in the retina is the photoreceptor layer, with secondary degeneration occurring later in retinal pigment epithelium (RPE) cells (De Sisternes *et al*, 2015; Yusuf *et al*, 2017). Some studies offer a potential explanation for this severe side effect.

By inhibiting the lysosomal degradation capacity and possibly endocytosis in RPE cells, HCQ and CQ are preventing the degradation of old and spent outer segments of photoreceptors in the RPE, a process that is required to maintain its function and preserve vision (Kevany & Palczewski, 2010; Yusuf *et al*, 2017). Furthermore, HCQ entrapment in the RPE might lead to an accumulation of lipofuscin, which is associated with photoreceptor function impairment and consequent vision loss (Kevany & Palczewski, 2010; Yusuf *et al*, 2017). It has been speculated that, due to this entrapment, retinopathy still continues in some cases after cessation of HCQ treatment (Michaelides *et al*, 2011). Accumulation of CQ in the pigmented ocular tissue, which comprises RPE cells, the iris, the choroid and the ciliary body, and eventually in the retina, was also observed in rhesus monkeys when CQ was administered for 52 months (Rosenthal *et al*, 1978). This caused an initial damage to the photoreceptors and the ganglion cells, followed by a disruption of both the RPE and choroid, which ultimately led to visual impairments and retinopathy (Rosenthal *et al*, 1978).

High levels of HCQ inhibit the function of the organic anion transporting polypeptide 1A2 (OATP1A2), a plasma membrane importer expressed in many tissues, including RPE cells (Xu *et al*, 2016). In particular, OATP1A2 transports all‐trans‐retinol (atROL), a retinol precursor essential for the classic visual cycle (Chan *et al*, 2015), into RPE cells. By blocking this transporter, HCQ causes an extracellular accumulation of atROL and disrupts the classic visual cycle (Xu *et al*, 2016).

### Cardiac side effects and myotoxicity

HCQ can cause acute and chronic cardiac adverse effects (Chatre *et al*, 2018). Acute adverse effects are linked to a very high dose of HCQ, which provokes a block of Na^+^ and Ca^2+^ channels. This inhibition can lead to membrane‐stabilization effects in cardiac muscle cells, which in turn causes conduction disturbances with atrioventricular block and QRS interval widening (White, 2007). Chronic adverse effects are connected to long‐term treatment with a high cumulative dose of HCQ (Chatre *et al*, 2018). As described above, HCQ treatment impairs the degradative activity of lysosomes, which leads to an accumulation of material such as glycogen and phospholipids in their interior (Chatre *et al*, 2018). In myocytes, this causes a vascularization of the cytoplasm and myofibrillar disorganization, which contributes to the development of cardiac myopathy and myocardial fibrosis (Yogasundaram *et al*, 2014). This phenomenon can also be seen in the Fabry and Danon lysosomal storage diseases, which have similar phenotypes (Roos *et al*, 2002; D'souza *et al*, 2014; Chatre *et al*, 2018). Moreover, HCQ‐mediated accumulation of autophagosomes in muscles and peripheral nerves can lead to myotoxicity or myotoxicity combined with peripheral nerve dysfunction (Shukla *et al*, 2019). Notably, HCQ and CQ also have proarrhythmic activity (Landmesser & Harrison, 2001; Khobragade *et al*, 2013; Chansky & Werth, 2017; Naksuk *et al*, 2020), which is of particular importance because of the potential use of this drug to treat COVID‐19 patients. These patients are burdened by arrhythmic events, and consequently, HCQ and CQ could worsen this pathological feature. It is still under investigation whether this proarrhythmic activity is caused by SARS‐CoV‐2 infection and whether HCQ and CQ are influencing it (Lazzerini *et al*, 2020).

## Conclusions

HCQ is nowadays widely used for the treatment of RADs and has shown great success in improving the quality of life of many patients. Over the years, research on the molecular and cellular mode of action of HCQ (and CQ) revealed that this compound modulates molecular processes and cellular responses in multiple ways. At least four mechanisms of action that, directly or indirectly, influence the immune system by synergistically dampening pro‐inflammatory responses, have been described. Although lysosomal inhibition and autophagy impairment are the most studied, HCQ also influences other important immune regulatory pathways by inhibiting specific steps, such as activation of endosomal TLR‐, cGAS and NOX signalling and Ca^2+^ mobilization for the ER. The beneficial therapeutic effect of HCQ in RADs probably lies in its multifaceted properties, which also makes it a promising candidate in other medical fields, such as oncology (Onorati *et al*, 2018) and microbiology (Savarino *et al*, 2003; Cortegiani *et al*, 2020; Yao *et al*, 2020).

Generally, HCQ is considered a safe drug with low prevalence of side effects. These side effects nevertheless exist and can impact the life of a patient tremendously. Among them, the most severe, i.e. retinopathy and cardiomyopathy, is linked to the induced lysosomal activity inhibition. This suggests that the unwanted negative effects of HCQ could be due to its lysosomotropic properties. In this context, it has been reported that the effect of HCQ on endosomal and lysosomal pH at therapeutic concentrations is negligible (Kužnik *et al*, 2011) and that the pH changes observed *in vitro* might not reflect the in vivo reality. Therefore, a higher dose of HCQ (or a higher cumulative dose) could lead to a pH increase in the compartments of the endolysosomal system and thus cause more side effects (Latasiewicz *et al*, 2017; Jorge *et al*, 2018). The well‐documented list of side effects caused by HCQ during the treatment of RADs should be considered when using HCQ to treat other pathologies such as cancer (Onorati *et al*, 2018), neurodegenerative disorders (Hedya *et al*, 2019), metabolic diseases (Pasquier, 2016) and microbial infections (Savarino *et al*, 2003), especially since treatment of some pathologies requires high HCQ doses (Leung *et al*, 2015).

While the search for a unifying mechanism of action for HCQ is tempting, current knowledge shows that this small molecule has more than a single target. As a result, future research should aim at identifying potential additional cellular and organismal pathways specifically modulated by HCQ. The mechanisms by which HCQ causes side effects could also provide important information. Increasing our understanding of HCQ mode of action would improve patient outcome by promoting therapeutic benefits while reducing side effects.

**Pending issues**

(i) Investigate whether all HCQ modes of action described with *in vitro* experiments are relevant in patients, and whether one of these mechanisms is predominantly causing the observed side effects.
(ii) Determine whether HCQ has other molecular effects than the ones described, which could help to better understand HCQ treatment outcomes in patients.
(iii) Chemically improve HCQ to make it more effective and less toxic, and thereby render it more suitable for the treatment of other diseases (e.g. specific cancers).
(iv) Understand how the anti‐inflammatory role of HCQ influences the anti‐viral and anti‐tumorigenic action of this drug in patients, and whether this could explain the observed discrepancies between the *in vitro* and *in vivo* results.

## For more information

(i) https://www.rheumatology.org/
(ii) https://www.sjogrens.org/
(iii) https://www.arthritis.org/
(iv) https://www.lupus.org/
(v) https://clinicaltrials.gov
(vi) https://www.fda.gov/

## Conflict of interest

The authors declare that they have no conflict of interest.

## Supporting information



Table EV1
