# Supplementary material for: Hydroxychloroquine in rheumatic autoimmune disorders and beyond
Source: EMBO Mol Med. 2020 Jul 26;12(8):e12476. doi: 10.15252/emmm.202012476 (PMC7411564; doi:10.15252/emmm.202012476)
Supplement: Supplementary file 1 — Table EV1 [file EMMM-12-e12476-s001.docx]

| **Table EV1: Summary of the documented side-effects of HCQ treatments** | | | |
| --- | --- | --- | --- |
| **Side effect** | **Condition** | **Comments** | **References** |
| **Ocular** | | | |
| Retinopathy | RA  SLE | **Prevalence:** In 7.5% of the patients after 5 years; in 20% of the patients after 20 years.  **Risk factors**: Doses > 5 mg/kg actual body weight, renal impairments, macular disease or use of tamoxifen.  **Clinical features:** Abnormalities in the retinal pigment epithelium, may later develop into parafoveal (bull’s eye) maculopathy or a pericentral pattern of damage. Ethnicity may play a role. May result in irreversible vision loss.  **Screening:** Most studies recommend to start after 5 years of HCQ use with annual check unless a risk factor is present.  **Treatment:** Cessation of HCQ | (Yusuf *et al*, 2017; Latasiewicz *et al*, 2017; Melles & Marmor, 2015; Forte *et al*, 2019; Kasturi, 2016; Generali *et al*, 2015) |
| Incidental retinal vascular occlusions | SLE | **Prevalence:** 2 cases. Severe vaso-occlusive retinopathy is rare.  **Risk factors:** Unknown  **Clinical features:** Visual loss.  **Screening:** SLE patients require prompt monitoring and evaluation throughout the treatment.  **Treatment:** Antiplatelet therapy could be initiated in patients with high risks to develop retinal vascular occlusion. Close ophthalmological monitoring and detection of early cases promotes good prognosis. | (Bajwa *et al*, 2015) |
| Corneal deposits | SLE  pSS  RA | **Prevalence:** Corneal deposits are observed in 5% of patients who take HCQ.  **Risk factors:** Unknown.  **Clinical features:** Asymptomatic or blurred vision and halos of colours around lights. Diplopia.  **Screening:** Presence of corneal deposits warrants closer follow-up.  **Treatment:** Unknown. | (Easterbrook, 1999) |
| **Cardiac** | | | |
| Conduction disorders | SLE  pSS  RA | **Prevalence**: Most common cardiac side effect.  **Risk factors:** High dosage, (renal, kidney) problems in the HCQ clearance.  **Clinical features:** Bundle or atrioventricular block, QTc prolongation and structural ECG abnormalities. Can occur acutely or chronically.  **Screening:** Electrocardiograms.  **Treatment:** Discontinuation of HCQ and treatment of the symptoms. | (Chatre *et al*, 2018; O’Laughlin *et al*, 2016; McGhie *et al*, 2018; Yogasundaram *et al*, 2014) |
| Myocardial disorders | SLE  pSS  RA | **Prevalence:** HCQ-induced cardiotoxicity is very rare.  **Risk Factors:** High dose, long drug administration periods, pre-existing cardiac disease and renal impairment, sex (females) and old age.  **Clinical features:** Left ventricular hypertrophy, ventricular hypokinesia, diastolic dysfunction, biventricular hypertrophy, cardiomyopathy with compensated heart failure and restrictive cardiomyopathy.  **Screening:** Unknown.  **Treatment:** Discontinuation of HCQ and treatment for heart failure. | (Chatre *et al*, 2018; Yogasundaram *et al*, 2014; Zhao *et al*, 2018; Dogar *et al*, 2018) |
| **Gastrointestinal** | | | |
| Acute HCQ-induced toxic hepatitis | SLE | **Prevalence:** 1 case.  **Risk factors:** Unknown.  **Clinical features:** Liver inflammation characterized by elevated liver enzymes, anorexia, nausea and non-specific upper abdominal pain.  **Screening:** Unknown.  **Treatment:** Cessation of HCQ is effective in controlling the condition. | (Abdel Galil, 2015) |
| Gastrointestinal (GI) upset | pSS  RA  SLE | **Prevalence**: GI intolerance is amongst the most common side effects of HCQ. It occurs in 10% of the patients.  **Risk factors:** Unknown.  **Clinical features:** Nausea, vomiting, diarrhea, anorexia, abdominal distension/ pain and transaminitis.  **Screening:** Unknown.  **Treatment**: Dose reduction. | (Wang *et al*, 2017; Rynes, 1997; Haładyj *et al*, 2018) |
| **Cutaneous** | | | |
| HCQ-induced erythroderma | SLE  RA | **Prevalence**: 2 cases.  **Risk** **factors**: An exacerbation of a pre-existing dermatoses (e.g. psoriasis, atopic dermatitis…).  **Clinical features:** Erythroderma with scales. Yellow toenails that showed hyperkeratosis with thickened nail plates.  **Screening:** Unknown.  **Treatment**: HCQ cessation and supportive/symptomatic treatment (monitoring hemodynamics status and temperature, correction of fluid and electrolytes imbalance, and nutritional support). | (Wang *et al*, 2018; Pai *et al*, 2017) |
| Longitudinal melanonychia | SLE | **Prevalence:** 1 case.  **Risk factors**: Easy bruising and SLE diagnosis.  **Clinical features:** Blueish gray longitudinal hyperpigmented bands on all nails with focal subungual haemorrhage.  **Screening:** Unknown.  **Treatment:** Suspension of HCQ but the changes might be irreversible. | (Zhang *et al*, 2019b) |
| Skin discoloration (grey-blue hyperpigmentation) | SLE | **Prevalence:** 7% of SLE patients, 50% of those cases occur in the first 5 years of treatment.  **Risk factors:** Bruising, corticosteroid use, oral anticoagulants, antiplatelet agents, antiphospholipid syndrome and skin trauma.  **Clinical features:** Macular and patchy blue-grey to black discoloration on the gums, palate, nails, face or arms, and invariably on the shins.  **Screening:** Unknown.  **Treatment:** HCQ cessation but complete clearance is rare. | (Coulombe & Boccara, 2017) |
| Cutaneous eruption | RA | **Prevalence**: Cutaneous manifestations are common in HCQ-treated patients.  **Risk factors:** Unknown.  **Clinical features:** Pruritus and rash.  **Screening:** Unknown.  **Treatment**: Dose reduction can relieve symptoms. | (Pearson *et al*, 2016) |
| **Neuromuscular** | | | |
| Elevated muscle enzymes (Creatine phosphokinase (CPK)) | SLE | **Prevalence**: 2/3 of patients on continuous HCQ treatment.  **Risk factors:** Unknown.  **Clinical features:** Elevated CPK enzyme. Can predispose to proximal muscle weakness or other myopathies.  **Screening:** Unknown.  **Treatment:** Unknown. | (Tselios *et al*, 2016) |
| Epileptic seizures | SLE | **Prevalence:** 1 case.  **Risk factors:** Previous mental illness.  **Clinical features:** Different seizure types have been noted e.g. complex partial epileptic seizure and tonic-clonic seizure.  **Screening:** Unknown.  **Treatment:** CQ cessation and treatment with an anti-convulsives. | (Krzeminski *et al*, 2018) |
| Myotoxicity | RA | **Prevalence:** Unknown.  **Risk factors:** May include Caucasian race and concomitant renal failure.  **Clinical features**: Affects proximal muscles; may be accompanied by peripheral neuropathy.  **Screening:** Unknown.  **Treatment:** Usually resolves with HCQ discontinuation. | (Haberl *et al*, 2005; Stein *et al*, 2000) |
| Neuromyopathy associated with skeletal muscle weakness | RA | **Prevalence**: 1 case.  **Risk factors:** Unknown.  **Clinical features:** Neuromyopathy and skeletal muscle weakness.  **Screening:** Unknown.  **Treatment**: HCQ discontinuation seems beneficial, as the condition is reversible. | (Yogasundaram *et al*, 2014) |
| **Neurologic** |  |  |  |
| Headache | SLE  RA  pSS | **Prevalence**: 10% or more patients display headache; 0.1% to 1% dizziness.  **Risk factors:** Unknown.  **Clinical features:** Headache/dizziness  **Screening:** Unknown.  **Treatment:** Unknown. | (Rynes, 1997) |
| **Hematologic** | | | |
| Relapsed HCQ-induced thrombocytopenia | SLE | **Prevalence:** 1 case.  **Risk factors:** Unknown.  **Clinical findings:** Accelerated destruction of platelets.  **Screening:** Unknown.  **Treatment:** HCQ cessation. | (Antón Vázquez *et al*, 2017) |
| Hemolysis | SLE | **Prevalence:** Uncommon.  **Risk factors:** Patients with glucose-6-phosphate dehydrogenase deficiency.  **Clinical features:** Unknown.  **Screening:** Testing is generally not considered necessary unless the patient has an underlying hematologic disease.  **Treatment:** Unknown. | (Antón Vázquez *et al*, 2017) |
| **Psychiatric** | | | |
| Exacerbation bipolar disorder | SLE | **Prevalence:** 1 case.  **Risk factors:** Unknown.  **Clinical features:** Exacerbation of manic and depressive episodes.  **Screening:** Unknown.  **Treatment:** HCQ cessation and psychiatric support can alleviate such exacerbations. | (Bogaczewicz *et al*, 2016) |
| Hypomnesia | SLE | **Prevalence:** 2 cases.  **Risk factors:** Unknown.  **Clinical features:** Progressive hypomnesis.  **Screening:** Unknown.  **Treatment:** HCQ cessation or reduction of its dose. | (Feng *et al*, 2018) |
| Anxiety and suicidal ideation | SLE  RA | **Prevalence:** Patients treated with HCQ show a higher percentage of anxiety and suicidal ideation.  **Risk factor:** High dose.  **Clinical features:** Generalized anxiety, suicidal ideation, self-harm, and auditory and kinaesthetic hallucinations.  **Screening:** Unknown.  **Treatment**: Adequate early psychiatric evaluation of patients treated with HCQ can be beneficial for early detection. | (Gonzalez-Nieto & Costa-Juan, 2015; Pinho De Oliveira Ribeiro *et al*, 2013) |
| HCQ-induced psychosis | RA | **Prevalence:** 1 case.  **Risk factors:** Unknown.  **Clinical features:** Auditory hallucinations, persecutory and partition delusions.  **Screening:** Unknown.  **Treatment:** HCQ cessation is effective in relieving the symptoms. | (Kwak *et al*, 2015) |
| **Other** |  |  |  |
| Ototoxicity (deafness) | SLE | **Prevalence:** Few cases reported.  **Risk factors:** Unknown.  **Clinical features:** Vestibular syndrome and bilateral perception deafness with hearing loss at 30 dB for frequencies starting at 1000 Hz. Also progressive unilateral hearing loss, tinnitus, and unilateral becoming bilateral.  **Screening:** Unknown.  **Treatment:** HCQ cessation. | (Chatelet *et al*, 2017) |
| Accidental HCQ-overdose | SLE  pSS  RA | **Prevalence:** The occurrence of HCQ overdose toxicity is rare, but it is potentially life-threatening.  **Risk factors:** Unknown.  **Clinical features:** Neurotoxic vestibulopathy, hypotension, hypokalemia-induced arrhythmia and death cases due to cardiac toxicity have been reported.  **Screening:** Unknown.  **Treatment:** HCQ cessation does improve the condition. Thus, symptomatic treatment and close cardiac monitoring of the patient is crucial. Correction of electrolyte imbalance and supportive measures are essential for improving the condition. | (Chansky & Werth, 2017) |
